# Quality Improvement Project to Improve Patient Satisfaction and Opportunity to Attend Clinical Team Meetings

**DOI:** 10.1192/bjo.2022.333

**Published:** 2022-06-20

**Authors:** Alec Rapson, Fiona Hynes, Aarohi Sharma, Talhah Malik, Lesley Beech, Caitlin Anderson, Ruhela Begum, Sima Naik

**Affiliations:** Birmingham & Solihull Mental Health NHS Foundation Trust, Birmingham, United Kingdom

## Abstract

**Aims:**

Clinical Team Meetings (CTM) are weekly multidisciplinary (MDT) meetings to review and discuss patients’ clinical care at Reaside Clinic, a medium secure inpatient forensic unit. Last year, there were significant difficulties in releasing nurses from ward duties to attend CTM, with effects on CTM efficiency and patients’ involvement and satisfaction in care. Furthermore, a hospital ‘protected mealtimes’ deadline commencing at 1230 meant additional pressure and further impacted patient attendance when meetings overran. The MDT (comprising doctors, nurses, psychologists, occupational therapists, pharmacists and admin staff) worked together to generate solutions, formulating a QI project to try to make improvements. Three primer drivers were: increasing patient satisfaction, increasing staff participation and increasing CTM efficiency.

**Methods:**

The MDT generated multiple change ideas to test to improve CTM experience and outcomes. Actions implemented included review of timetabling of patients to ensure adequate timekeeping and ensuring availability of attending ward staff, defining patient expectations from CTM through increased communication, and seeking patient feedback on satisfaction or engagement after each CTM through anonymous questionnaires. Data were collected at CTMs from February to April 2022, retrospectively compared to reference data collected before QI actions were implemented. A Microsoft OneDrive document was shared between the MDT to ensure accurate data collection, with information collected on CTM finish time, number of patients offered to attend, number of patients who did attend and anonymous patient satisfaction feedback from questionnaires.

**Results:**

Early indications show improvement in meeting timelines and increased staff satisfaction with the CTM process, with data collection ongoing. Baseline results from September 2021 show an average of only 2 of between 13–15 patients attending weekly, in addition to finishing beyond the 1230 target on almost all occasions. Anecdotal evidence from the MDT showed poor patient satisfaction and engagement with the process before QI changes were implemented. Full results will be available by the time of presentation; currently, an average of 4 patients have attended CTM each week with all sessions finishing on time since implementation of changes.

**Conclusion:**

Patient involvement in care and person-centred care are key to improving engagement and satisfaction with inpatient psychiatric management in forensic settings. Targeted multiple change ideas implemented by the MDT through this QI aim to improve patient satisfaction through enabling increased opportunity to attend weekly CTM, with modifications to the CTM process from key staff. Preliminary results show increased opportunity of patients to attend CTM, increased staff and patient satisfaction, and increased CTM efficiency.

